# Peritoneal Protein Losses and Cytokine Generation in Automated Peritoneal Dialysis with Combined Amino Acids and Glucose Solutions

**DOI:** 10.1155/2007/97272

**Published:** 2007-12-17

**Authors:** H. L. Tjiong, F. J. Zijlstra, T. Rietveld, J. L. Wattimena, J. G. M. Huijmans, G. R. Swart, M. W. J. A. Fieren

**Affiliations:** ^1^Department of Internal Medicine, Erasmus MC, 3015 CE Rotterdam, The Netherlands; ^2^Department of Anaesthesiology, Erasmus MC, 3015 CE Rotterdam, The Netherlands; ^3^Department of Clinical Genetics, Erasmus MC, 3015 CE Rotterdam, The Netherlands

## Abstract

*Objectives*. Protein-energy malnutrition as a consequence of deficient protein intake frequently occurs in peritoneal dialysis (PD) patients. Previously, we showed that peritoneal dialysate containing a mixture of amino acids (AA) and glucose has anabolic effects. However AA-dialysate has been reported to increase intraperitoneal protein and AA losses and the release of proinflammatory cytokines (interleukine-6 (IL-6) and tumor necrosis factor alpha (TNFα)). We investigated the effect of AA plus glucose (AAG) solutions on peritoneal protein losses and cytokine generation. *Methods*. In 6 patients on standard automated peritoneal dialysis (APD) 12 APD sessions of 6 cycles each were performed during the night using dialysate containing 1.1% AA plus glucose or glucose alone as control. Protein losses and TNFα and IL-6 concentrations were measured in dialysates separately collected from nightly cycling and daytime dwell. *Results*. The 24 hour-protein losses with AAG (median 6.7 g, range 4.7–9.4 g) were similar to control dialysate (median 6.0 g, range 4.2–9.2 g). Daytime dialysate IL-6 levels were higher after nightly AAG dialysis than after control dialysis (142 pg/ml and 82 pg/ml, respectively, P<.05). TNFα concentrations were very low. *Conclusion*. Nightly APD with amino acids containing dialysate was associated with an increase in peritoneal IL-6 generation during the day. The addition of AA to standard glucose dialysis solutions did not induce a significant increase of peritoneal protein losses.

## 1. INTRODUCTION

One of the untoward effects of peritoneal dialysis (PD) is the loss of proteins and amino acids into the dialysate effluent. Patients undergoing continuous ambulatory peritoneal dialysis (CAPD) lose amino acids, 2–4 g a day, and a substantial amount of proteins, varying between 5–15 gram per day. The protein and AA losses increase during peritonitis [1–9]. The continuous loss of proteins and amino acid through peritoneal clearance can be compensated by adequate dietary intake, but a poor appetite limits protein intake in many patients [10]. Previously we showed that the gain in protein provided by supplying amino acids (AA) plus glucose (G) intraperitoneally exceeds the usual 24 hours protein and AA losses through peritoneal clearance [11]. However, it has been reported that AA-based dialysate may increase intraperitoneal protein losses, as well as stimulating the release of various vasoactive substances including several proinflammatory cytokines [12–15].

In this study we investigated the protein losses into overnight and daytime dialysate effluents and changes in the levels of the cytokines TNFα and IL-6 in effluent and in plasma, comparing dialysate containing AA plus G (AAG) with glucose (G) only solutions in patients on APD. Additionally we determined the peritoneal AA losses into the overnight and daytime effluents using standard glucose-based dialysis solution.

## 2. PATIENTS AND METHODS

### 2.1. Patients

Six patients who had been on APD for more than 3 months and had a weekly Kt/V greater than 1.7 were recruited from the Peritoneal Dialysis Unit of the Erasmus Medical Center (Rotterdam, The Netherlands). Patients who had had peritonitis or other infectious or inflammatory diseases in the previous 6 weeks, a malignancy, or a life expectancy *<* 6 months were excluded. The Medical Ethical Committee of Erasmus MC approved the study and written informed consent was obtained from all patients.

### 2.2. Study design

This study was a part of our previous open label, randomized, crossover single center study of the effects of dialysate containing AA plus G (Nutrineal 1.1% plus Physioneal 1.36–3.86% G (Baxter BV, Utrecht, the Netherlands) on whole body protein metabolism, compared with a control solution containing G alone (Physioneal 1.36%–3.86%). Prior to the study all patients were treated with standard glucose-based dialysis fluid (Dianeal or Physioneal, Baxter BV, Utrecht, The Netherlands). After the patients were randomized to start with either AAG or with G (concentration of 1.36%–3.86% depending on the ultrafiltration targets) each fluid was used in two consecutive periods of 7 days. The APD schedule for each patient before and during the study was kept constant.

The dialysis procedure during the study was as follows. Six nighttime exchanges were performed automatically using a cycler (HomeChoice, Baxter BV). In the daytime, all patients, except for one patient, were treated with polyglucose-containing dialysate (Extraneal, Baxter BV). Two patients had an additional exchange with glucose solution (Physioneal). The APD schedule for each patient was similar to that used before the study in order to meet adequacy and ultrafiltration targets. The cycler regulated the mixing procedure. The AA 1.1% solution contained as buffer 40 mmol/L lactate and the 1.36%, 2.27%, or 3.86% G-solutions 25 mmol/L bicarbonate, plus 15 mmol/L lactate.

At the end of each study week after an overnight fast using either AAG or G only dialysate and before the morning exchange, venous blood samples were taken for determination of the levels of C-reactive protein and the cytokines, interleukine-6 (IL-6), and tumor necrosis factor alpha (TNFα). In all 6 patients overnight and daytime dialysates were collected separately during the last three days of each week to determine cytokine and protein and amino acid concentrations.

### 2.3. Analytical determinations

#### 2.3.1. Blood chemistry

Concentrations of C-reactive protein and dialysate protein were determined by particle-enhanced immunoturbidimetric assay. The dialysate amino acids were analysed by ion exchange chromatography on a Biochrome 20 aminoacid analyser (Biochrom, Cambridge, UK) with UV-V is detection using ninhydrin.

#### 2.3.2. Cytokines

For measurement of TNFα and IL-6, plasma and dialysis fluid samples were diluted 1 : 1 in appropriate calibrator diluent assay buffer. Cytokine assays were performed following the manufacturers protocol (Pelikine human ELISA compact kits for IL-6 (cat. no. M1906) and TNFα (cat. no. M1920), Sanquin, Amsterdam, the Netherlands). The standard curve ranges and mean calculated zero signal plus 3 SD for IL-6 were 0–450 pg/mL and 0.2 pg/mL, respectively; and for TNFα 0–1000 pg/mL and 0.5 pg/mL, respectively. The requested solutions were provided with the ELISA compact kits and additional toolkits (Pelikine-Tool set (cat. no. M1980)). Following the manufacturers assay instruction step by step, at the end of the procedure the absorbance per well was measured at 450 nm with a Medgenix ELISA reader. Sample concentrations were calculated using the appropriate standard calibration lines and the Softmax software of the reader.

## 3. STATISTICAL ANALYSIS

Data were analyzed using the statistical program SPSS, version 14.0, for Windows (SPSS Inc., Chicago, Ill, USA). Data are expressed as median with range. Comparisons of outcomes were made using the Wilcoxon Signed Rank test. All tests of significance were two-sided, and differences were considered statistically significant when the *P* value was <.05.

## 4. RESULTS

Table 1 shows the baseline characteristics of the patients, including the peritoneal membrane characteristics as measured by peritoneal equilibrium test (PET) [11]. Three patients were anuric. Apart from the use of medication regularly taken by PD patients, two patients used prednisone in a maintenance dose. Peritoneal ultrafiltration after administration of AAG-based dialysis fluid, determined as the difference between dialysate inflow and outflow, did not differ from that with G only dialysate. Serum C-reactive protein levels ranged from 6–65 mg/L (median 7.5 mg/L) with AAG-based dialysate and from 1 to 33 mg/L (median 9.5 mg/L) using G only dialysate. The protein losses per 24 hours, that is, daytime dwell plus overnight APD with standard G solution ranged from 4.2 to 9.2 g (median 6.0 g) were not significantly different from that occurring with AAG, which ranged from 4.7 to 9.4 g (median 6.7 g). As shown in Table 2, there was no difference in protein losses between the dialysates. The appearance rate of protein losses in the overnight effluent was significantly higher than that in the daytime. The mean total AA losses, that is, essential AA (EAA) and nonessential AA (NEAA) per 24 hours, that is, daytime dwell plus overnight APD with standard G solution varied between 1.7 and 3.2 g (median 2.2 g), and 26 ± 1.7% of dialysate AA were essential AA. Using standard G only dialysis solution, the total AA losses in the 8.5 hour-overnight effluents were higher than in the 15.5 hour-daytime effluents. Likewise, the appearance rate of total AA losses into overnight dwells was significantly higher than that into daytime dwells (Table 2). The total EAA losses in the overnight dwell were also higher than that in daytime dwell. In particular, IL-6 levels in daytime effluents were significantly higher than those in plasma (Table 3). Dialysate IL-6 concentrations at daytime dwell differed significantly from overnight dwell during both type of dialysis schemes. The IL-6 levels in the daytime effluents after nightly cycling with AAG were significantly higher than that after G. Plasma and both overnight and daytime effluent levels of tumor necrosis factor α (TNFα) were low using both AAG and G-only dialysis solutions.

## 5. DISCUSSION

As part of previous work on the effects of amino acids containing dialysate on whole body protein metabolism, we investigated in this study the loss of proteins in dialysate in APD patients using either the standard dialysate containing only glucose or a mixture of amino acids and glucose. In addition, we studied the effects of these dialysis fluids on the peritoneal release of the cytokines IL-6 and TNFα and we investigated the losses of amino acids with the use of glucose-based (standard) dialysis solution. Although APD is widely used, the peritoneal protein losses have been studied only occasionally in adult patients undergoing APD.

We found that the mean protein losses per 24 hours, that is, the sum of daytime dwell and nightly APD with standard glucose dialysate was on average about 6 grams, which is in the same order of magnitude as previously reported for CAPD patients by various authors [3, 4]. The peritoneal protein losses during 8.5 hour nightly APD and in the daytime dwell of 15.5 hour were similar. By comparison, in a recent study, protein losses during nightly cycling were found to be rather high, exceeding protein losses during the daytime dwell [16]. Yet, also in our patients protein losses per time were higher during cycling with standard glucose solution. This may seem surprising as it is generally accepted that peritoneal protein losses, in contrast to small molecular weight solutes, are linear with time irrespective of the number of dialysate exchanges. It has, however, been shown in patients on intermittent peritoneal dialysis (IPD) that protein loss is greater during the first dwell after a “dry” period and stabilizes at lower levels after a few exchanges. This finding could be explained by wash out of proteins from the previous dry period [17, 18]. Assuming a residual volume of 400–500 mL in our patients, wash out of proteins from the long daytime dwell could account for the difference of protein losses per time between day time dwell and nighttime cycling.

In previous studies we showed that the amino acids plus glucose containing dialysis fluid has anabolic effects on protein metabolism [11, 19]. The current study shows that the presence of amino acids in the dialysate did not increase protein loss in the overnight dialysate effluent as compared with G only dialysis solutions in APD patients. Others have reported that a 2.6% amino acid peritoneal dialysis solution induced an increased loss of macromolecules including albumin and IgG and the small molecular weight amino acids, which was accompanied by an increased prostanoid generation in the peritoneal cavity [12]. One percent AA solutions were also found to stimulate protein losses and release of prostanoids and several proinflammatory cytokines consistent with an increase in peritoneal blood flow and effective peritoneal surface area [13, 14]. In some studies, however, no significant effect on protein losses or release of prostanoids was found [20, 21].

Reports on the peritoneal losses of AA in APD are scarce. In our present study the mean 24-hour AA losses, that is, daytime dwell and nightly APD were similar as reported earlier in patients who are on CAPD and somewhat higher compared with a previous report in APD patients, using standard G dialysis solution [1, 2, 16]. We found that total AA losses were greater during nightly APD than during the long daytime dwell with standard G-based dialysis solution. This finding is in line with the fact that transperitoneal transport of small molecular weight solutes is dependent on the dialysate flow rate, that is, number of dialysate exchanges. Approximately 26% of the effluent AA were essential AA in agreement with previous findings in patients on CAPD [1].

We also studied the influence of amino acid-based dialysate on cytokines both during the night- and at daytime. The levels of TNFα and IL-6 as found in the present study in daytime dialysate are on the whole comparable to those described in several studies in CAPD patients with standard glucose dialysis solutions during an infection-free period [13, 22–24]. Daytime dialysate IL-6 levels were significantly higher when during the preceding nightly APD dialysate was used that contained amino acids instead of only glucose. This finding may be somewhat puzzling as during nightly cycling no statistically significant difference in IL-6 levels was found. It cannot be ruled out, however, that any effect of AA on IL-6 during cycling was diluted by rather low IL-6 concentrations as a result of the high dialysate flow rate. It has been shown in several studies in CAPD that amino acid dialysate was accompanied with increased levels of various cytokines including IL-6 and TNFα [13, 25]. There is ample evidence that even in the absence of peritonitis IL-6 is produced locally within the peritoneal cavity rather than transported across the peritoneal membrane. Our finding that IL-6 levels in dialysate were higher than in plasma, especially during the long daytime dwell, are consistent with local production and release [22, 23]. The main source of peritoneal TNFα is thought to be the mononuclear phagocyte, whereas both mesothelial cells and macrophages are able to produce and release substantial amounts of IL-6 [23, 26]. The release of IL-6 from mesothelial cells occurs constitutively and can be stimulated by macrophage-derived TNFα and IL-1β [13,23,26]. In the dialysate of patients with PD-related peritonitis and in ascites of liver cirrhosis patients with spontaneous bacterial peritonitis, high levels of various cytokines including TNFα and IL-6 are found due to increased local production [23, 26–28]. In the present study, the patients were clinically free of infection, and C-reactive protein levels in most of the patients were normal or only slightly increased. Dialysate TNFα was very low both with the standard glucose and the amino acid and glucose mixture. The clinical implications of increased intraperitoneal IL-6 levels are far from straightforward. Any increases in levels of cytokines in dialysate in vivo may be interpreted as a sign of a local inflammatory stimulus or as an improvement of the capacity to synthesize inflammatory factors. It is therefore a matter of debate as to whether any changes in cytokine release should be considered as harmful or as a marker of improved tissue responsiveness.

In conclusion, our study suggests that in APD the use of dialysis fluid containing a mixture of amino acids and glucose induces an increase in peritoneal production of IL-6 as compared to dialysate containing only glucose. In contrast, no appreciable amounts of TNFα were found in peritoneal dialysate. This is the first controlled study to investigate the effects of amino acids containing dialysate on cytokines and protein losses. A limitation of this study is the small number of patients. Further studies are required to elucidate the relationship between amino acids containing dialysate and the cytokine network of the peritoneal cavity.

## Figures and Tables

**Table 1:** Baseline characteristics of the patients^a^.

Patient	Primary diagnose of renal disease	Time on PD (months)	Gender	Age (years)	BMI (wt/ht^2^)	Kt/V (weekly)	PET
1	Nephrosclerosis	14	M	57	23.9	1.95	HA
2	Reflux nephropathy	8	M	43	25.1	1.85	HA
3	Alport disease	63	M	35	25.9	1.82	H
4	Rapidly progressive glomerulonephitis	45	M	56	22.8	2.04	H
5	Periarteritis nodosa	5	M	47	22.1	1.76	HA
6	Polycystic disease	36	F	45	30.1	1.98	HA

Mean		29		47	25	2	
SD		23		8	3	0.1	

^a^M, male; F, female; BMI (wt/ht^2^), body mass index (weight/height^2^); PD, peritoneal dialysis; Kt/V is the urea clearance adjusted for distribution volume, using PD adequest 2.0, software Baxter^11^; PET, peritoneal equilibrium test; H, high; HA, high average.

**Table 2:** Protein and amino acid losses in dialysis effluent in APD patients^a^.

	Overnight		Daytime	
	AAG dialysis (1)	G dialysis (2)	After AAG dialysis (3)	After G dialysis (4)
Protein (g/8.5 h)	3.06 (2.28–3.51)	3.0 (1.84–3.99)	3.48 (2.44–6.16)	3.03 (2.17–5.54)
Protein (g/h)	0.36 (0.27–0.41)	0.35 (0.22–0.47)^b^	0.22 (0.16–0.40)	0.20 (0.14–0.36)
Total AA (g/8.5 h)		1.47 (1.02–1.94)^c^		0.70 (0.62–1.25)
EAA (g/8.5 h)		0.37 (0.25–0.55)^d^		0.18 (0.17–0.37)
Total AA (g/h)		0.17 (0.12–0.23)^e^		0.05 (0.04–0.08)

^a^Data are expressed as median and range; AAG, amino acid and glucose; G, glucose.

Comparisons were made for columns (2) versus (4). These resulted in significant differences for: b, c, d, and e (*P* *<* .05 versus daytime after G).

No significant differences were found for columns (1) versus (2) and (3) versus (4).

**Table 3:** IL-6 and TNFα levels in plasma and dialysate^a^.

			Overnight		Daytime	
	Plasma AAG (1)	Plasma G (2)	Dialysate AAG (3)	Dialysate G (4)	After dialysate AAG (5)	After dialysate G (6)
IL-6 (pg/mL)	13 (4–26)	13 (3–48)	21 (16–59)	17 (13–40)	142 (71–599)^b,d,f^	82 (51–338)^c,e^
IL-6 (ng)			247 (206–784)	197 (168–536)	343 (240–1613)	178 (133–872)
TNFα (pg/mL)	2 (1–52)	2 (1–49)	1 (1–1)	1 (0–2)	2 (1–39)	2 (1– 5)
TNFα (ng)			15 (9–18)	14 (5–21)	5 (2–93)	5 (2–12)

^a^Data are expressed as median and range. AAG, amino acid and glucose; G, glucose; IL-6, interleukine-6; TNFα, tumor necrosis factor α.

Comparisons for IL-6 (pg/mL) were made for columns (1) versus (5), (2) versus (6), (3) versus (4), (5) versus (6), (3) versus (5), (4) versus (6), and (5) versus (6).

These resulted in significant differences for b (*P* *<* .05 versus plasma AAG); c (*P* *<* .05 versus plasma G); d (*P* *<* .05 versus overnight dialysate AAG); e (*P* *<* .05 versus overnight dialysate G); f (*P* *<* .05 versus daytime after dialysate G).

No significant differences were found for columns (1) versus (3) and (2) versus (4).
